# Influence of Infill Patterns on the Shape Memory Effect of Cold-Programmed Additively Manufactured PLA

**DOI:** 10.3390/polym16172460

**Published:** 2024-08-29

**Authors:** Vladimir Barrera-Quintero, Erasmo Correa-Gómez, Alberto Caballero-Ruiz, Leopoldo Ruiz-Huerta

**Affiliations:** 1 Programa de Maestría y Doctorado en Ingeniería, Universidad Nacional Autónoma de México (UNAM), Edificio “T-Bernardo Quintana Arrioja”, Primer Piso, Ciudad Universitaria, Mexico City 04510, Mexico; vladimir.barrera@icat.unam.mx; 2 Instituto de Ciencias Aplicadas y Tecnología (ICAT), Universidad Nacional Autónoma de México (UNAM), Circuito Exterior S/N, Ciudad Universitaria, Mexico City 04510, Mexico; erasmo.correa@icat.unam.mx (E.C.-G.); alberto.caballero@icat.unam.mx (A.C.-R.); 3 Programa Investigadoras e Investigadores por México—Consejo Nacional de Humanidades, Ciencias y Tecnologías CONAHCYT, Benito Juárez, Mexico City 03940, Mexico; 4 National Laboratory for Additive and Digital Manufacturing, MADiT, Mexico

**Keywords:** four-dimensional additive manufacturing, 4DAM, shape memory effect, cold programming, infill pattern, PLA, digital manufacturing, material extrusion

## Abstract

In four-dimensional additive manufacturing (4DAM), specific external stimuli are applied in conjunction with additive manufacturing technologies. This combination allows the development of tailored stimuli-responsive properties in various materials, structures, or components. For shape-changing functionalities, the programming step plays a crucial role in recovery after exposure to a stimulus. Furthermore, precise tuning of the 4DAM process parameters is essential to achieve shape-change specifications. Within this context, this study investigated how the structural arrangement of infill patterns (criss-cross and concentric) affects the shape memory effect (SME) of compression cold-programmed PLA under a thermal stimulus. The stress–strain curves reveal a higher yield stress for the criss-cross infill pattern. Interestingly, the shape recovery ratio shows a similar trend across both patterns at different displacements with shallower slopes compared to a higher shape fixity ratio. This suggests that the infill pattern primarily affects the mechanical strength (yield stress) and not the recovery. Finally, the recovery force increases proportionally with displacement. These findings suggest a consistent SME under the explored interval (15–45% compression) despite the infill pattern; however, the variations in the mechanical properties shown by the stress–strain curves appear more pronounced, particularly the yield stress.

## 1. Introduction

Additive manufacturing (AM) is defined by ISO/ASTM 52900:2021(E) [1] as the process of joining materials to produce parts from 3D model data, usually layer upon layer, as opposed to subtractive manufacturing and formative manufacturing methodologies. AM is classified into seven processes: binder jetting, directed energy deposition, material jetting (MJ), powder bed fusion (PBF), sheet lamination, vat photopolymerization (VP), and material extrusion (ME) [1].

Four-dimensional additive manufacturing (4DAM), also known as “4D printing”, is a process that integrates the components fabricated by AM processes with time. These can respond by changing their state (shape or performance [2,3,4,5]) when a stimulus is applied (heat, humidity, electric and magnetic fields, etc.) [6,7,8,9]. This inherent responsiveness enables them to adapt and perform specific functions. The main AM processes used in 4DAM are MJ, VP, PBF, and ME [10].

In particular, ME is the process in which material is selectively dispensed through a nozzle or orifice [1]. Multiple rasters form a layer, and parts are formed by stacking layers. ME involves several process parameters (PPs) that significantly impact the properties of the produced components. These parameters include layer thickness, feed rate, build orientation, infill density and pattern, raster size, deposition speed and orientation, nozzle temperature, and build platform temperature [11,12]. PPs influence the bonding between rasters and layers, ultimately affecting the part’s mechanical performance [11]. The influence of PPs on the mechanical properties has been widely studied [13,14,15,16,17,18]. The infill pattern (IP) is a particularly crucial parameter because it directly affects the arrangement of rasters and pores within the inner structure [11]. This arrangement defines the “filling strategy” within the part. Due to the inherent raster and pore layout, ME parts exhibit a periodic porous structure [19]. Even seemingly simple geometries under load can experience complex internal stress states. This complexity arises from the size, shape, and distribution of pores within the structure, ultimately impacting the mechanical behavior of the part [19].

Several IPs have been investigated for the ME process, including linear, grid, rectilinear, triangular, trihexagon, hexagonal, cubic, cubic subdivision, quarter cubic, cross, honeycomb, and concentric patterns [20,21,22,23]. Studies by Prajapati et al. [24] using rectilinear, concentric, and Hilbert curve IPs revealed that both rectilinear and concentric patterns achieved the highest compression strength. Akhoundi et al. [23] compared concentric, rectilinear, Hilbert curve, and honeycomb IPs at 20%, 50%, and 100% infill density and evaluated their impact on the mechanical properties. The concentric pattern resulted in the highest tensile and flexural strengths, likely due to the alignment of the rasters with the loading direction. Additionally, higher infill percentages yielded greater tensile and flexural strengths and moduli. To predict the mechanical properties of ME parts considering their internal structure, Sánchez-Balanzar et al. [19] performed a computational multiscale homogenization analysis of unidirectional and criss-cross IPs. They found good agreement between the analysis results and experimental data, particularly regarding Young’s modulus, which varied significantly between the studied IPs, especially in the upright build direction.

Building on the established influence of both PPs and IP on mechanical properties, incorporating thermoplastic shape memory polymers (TSMPs) into AM introduces an additional factor: the ability to alter the shape memory effect (SME) [25]. TSMPs are widely used [26,27]. They have the capability of storing a temporary shape by thermomechanical programming (TMP) and recovering their original shape by applying a thermal stimulus; this effect is called SME [28]. Moreover, there are other 4DAM studies [29,30,31] in which components that do not require programming to respond to a stimulus are presented. After being fabricated, they are able to respond immediately when a stimulus is applied. In this case, several studies have been conducted to analyze the influence of PPs on the component response [29,30,31]. Two characteristics are commonly used to evaluate their shape memory effect: the shape fixity ratio (SFR) and the shape recovery ratio (SRR). These characteristics measure the capability to store a temporary shape and recover its original shape [32,33].

**Figure 1:**
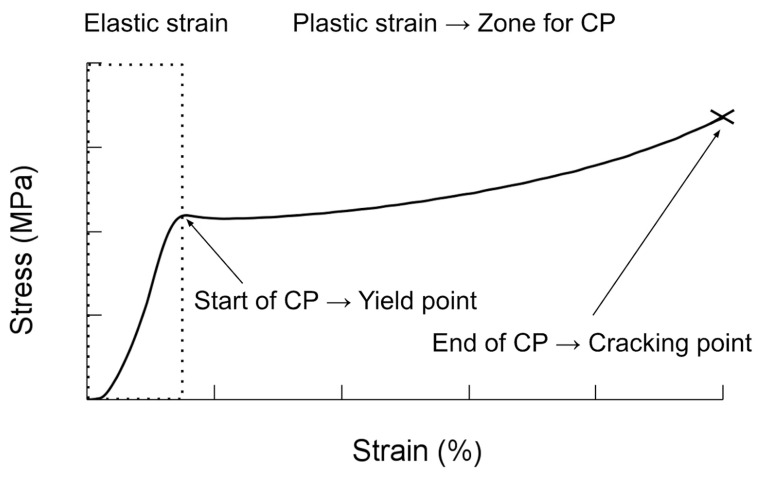
Stress–strain curve for cold programming.

TMP can be realized at different temperatures through hot, warm, and cold programming (HP, WP, and CP, respectively) [34,35]. HP requires heating the material to above its glass transition temperature (Tg); then, loading is applied to deform it. To store the temporary shape, it must be cooled below the Tg under load; then, the load is withdrawn and the temporary shape is fixed. To recover its original shape, it is heated to above the Tg. WP is very similar to HP, but the material is heated at the Tg and then loading is applied to deform it. To store the temporary shape, it must be cooled below the Tg under load; then, the load is withdrawn, and the temporary shape is fixed. To recover its original shape, it is heated above the Tg. For CP, the material is deformed at a temperature below the Tg. Unlike in the two previous processes, irreversible deformation or plastic deformation is required to program the temporary shape (Figure 1) [36,37]; however, the applied deformation should be lower than the cracking point [38]; then, recovery is performed through heating above the Tg. 

CP offers several advantages compared to HP and WP [37,39,40], such as a simple programming process (loading at low temperatures to stabilize the desired temporary shape), energy and time savings (no time passes and no energy is spent on heating), partial reversible plastic deformation, higher recovery stresses than HP, and faster recovery than HP. On the other hand, it requires higher stresses for programming and shows higher values of elastic springback [40]. Several studies have modified polymers for cold programming, or have improved their performance through blending with different polymers, or have added additives and carbon nanotubes [41,42,43,44]. Other aspects of CP, such as mathematical modeling [36,45,46], the influence of the conditions under which CP is realized [2,40,47,48], and structural changes in the material for CP, have also been studied [49]. 

Experimental results based on the effect of the layer thickness and raster angle on the ME process, considering HP [25], showed that the layer thickness exerted the smallest effect on the SRR, and the raster angle exerted the minimum effect on the time taken for shape recovery. Nevertheless, the SME depended strongly on the recovery temperature. Nonetheless, a gap exists in the understanding of how PPs modify the SME. This understanding will allow designers of 4DAM components to take advantage of not only the material and geometry characteristics but also the PPs to be included in the SME. 

This paper aims to contribute to the field of 4DAM by investigating the influence of IPs on CP for additively manufactured components with SME capabilities. Specifically, this research tracked changes in the SFR and SRR of samples made of PLA, fabricated using the ME process and programmed under compression conditions. The obtained results are expected to be valuable for incorporating IP parameters into the design process of 4DAM components, thus providing additional considerations for modifying the SME. 

## 2. Materials and Methods

**Figure 2:**
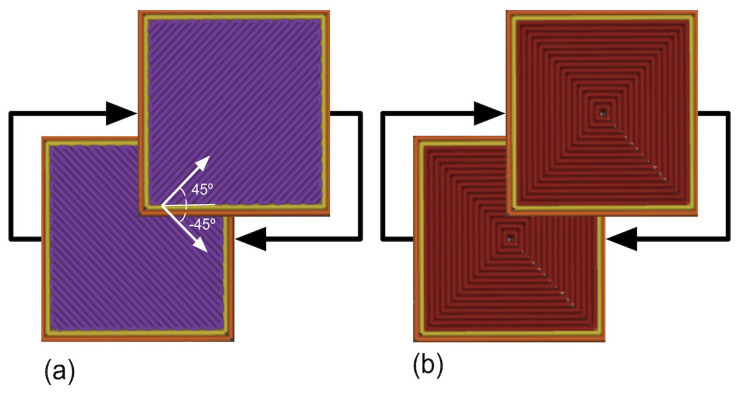
Infill patterns proposed: (**a**) criss-cross and (**b**) concentric patterns.

Concentric and criss-cross (−45/45) IPs (Figure 2) were employed, because they are known to significantly influence the mechanical properties of AM components [19,23,24]. Seven cubic samples of 12.7 mm were manufactured using ME on a Creatbot^®^ F160 machine and sliced through Creatware^®^ software (version 7.0.2) for each IP. The manufacturing PPs employed are presented in Table 1. Once manufactured, the samples were then cold-programmed at 20 °C under compression conditions. After unloading, the SFR was measured. A thermal stimulus of 60 °C was chosen for this study because, above a Tg of 55 °C (as specified on the PLA-filament technical data sheet from a DSC analysis according to Creatbot^®^), the structure relaxes to its equilibrium configuration, leading to shape recovery [46,50,51]. The SRR was measured after application of the stimulus. The results presented in Section 3 were obtained from the average of the seven samples with the corresponding standard deviation (SD). 

**Figure 3:**
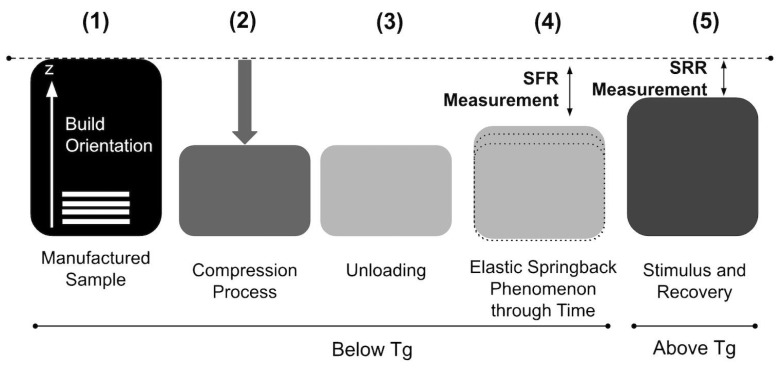
Experimental methodology.

The experimental process for both IPs involved the following steps: (1) sample manufacturing, (2) compression, (3) unloading, (4) elastic springback, and (5) recovery. Figure 3 illustrates this process.

## 3. Results and Discussion

### 3.1. Compression and Unloading

**Figure 4:**
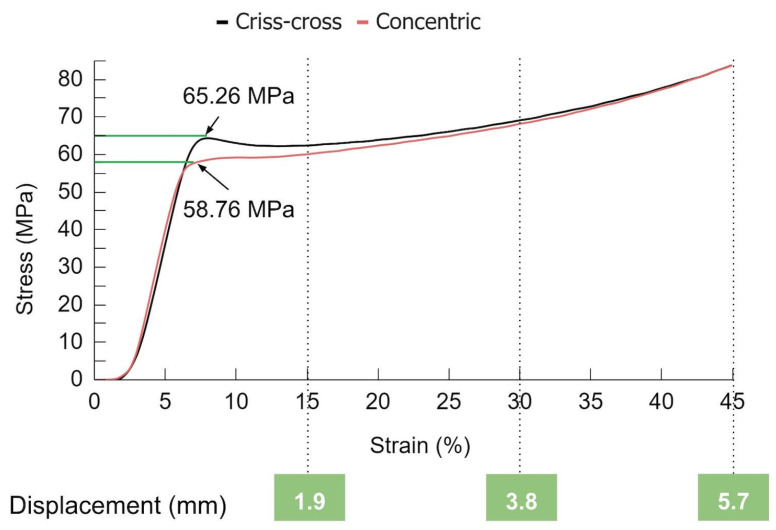
Stress–strain response during compression testing for samples deformed to 15%, 30%, and 45% strain.

The deformation of the cubic samples was achieved using a Universal Testing Machine (Shimadzu AG-X 50 kN, Kyoto, Japan) in compression mode at 20 °C. The load direction was aligned with the build orientation for all cases. The data were collected with TRAPEZIUM LITE X software (version 1.5.0c). A controlled compression test was programmed with a 50 kN load cell. The compression speed was set to 1.3 mm/min, according to ASTM D695 [52]. To ensure proper contact, a preload of 2 N was applied to the samples. The experiment then proceeded with different compressive strain percentages: 15%, 30%, and 45%. These strain percentages corresponded to displacements (*D_d_*) of 1.9 mm, 3.8 mm, and 5.7 mm, respectively, all located within the plastic zone. After the displacement was achieved, the load was withdrawn immediately. Figure 4 shows the stress–strain diagram obtained during compression.

It is possible to observe that the influence of the IP on the stress–strain diagrams was mainly due to yielding, and the results tended to be quite similar. Although the yielding point is a characteristic that depends on the temperature and strain rate [53], it could also be modified through the PPs, as can be observed. The criss-cross pattern shows elastic, yield, softening, and hardening stages. However, the concentric pattern curves do not show a softening stage in the stress–strain curve. The yield point occurred at 65.26 ± 0.88 MPa and 58.76 ± 0.89 MPa for the criss-cross and concentric patterns, respectively. For the concentric samples, yielding began at a smaller strain and required less stress. Additionally, the stress required to reach the desired displacement was lower for the concentric pattern in all cases.

Studies [23,24] showed that the stress–strain curve is affected by the type of IP due to the bonding between the rasters, the quantity and shape of the voids or gaps, and the direction in which the load is applied to the component. IPs affect stress–strain curves. Figure 4 shows that criss-cross pattern likely absorbs more energy elastically due to its load distribution, as was studied and validated for both IPs by Sánchez-Balanzar [19,23,24].

### 3.2. Elastic Springback

After compression, and immediately after the load is withdrawn, the samples exhibit an “elastic springback,” a strain recovery that changes over time, influenced by the type of polymer [36,40,54], type of TMP, conditions under which programming was performed [46], and PPs. To calculate the springback (*E_s_*) after compression, subtract the desired displacement (*D_d_*, e.g., 1.9 mm, 3.8 mm, 5.7 mm) from the initial height (*H_i_*, 12.7 mm) of the sample. Then, subtract this value from the final compressed height (*H_es_*) after 3 h when no further springback occurred, Equation (1).

(1) $$E_{s}\left(mm\right)=H_{es}\;\left(mm\right)-\left(H_{i}\;\left(mm\right)-D_{d\;}\left(mm\right)\right)$$

Table 2 presents the elastic springback measured at different displacements for both IPs. For both IPs, as the displacement increased, the amount of elastic springback also increased. However, the percentage of springback relative to the total deformation decreased. The criss-cross pattern exhibited the highest elastic springback (0.99 mm). 

According to other studies, elastic springback can be minimized by increasing the stress relaxation time (the time at which the load is maintained before removing it) or structural relaxation time (the period after unloading), but the programming time increases. Shahi et al. [37] decreased the elastic springback by increasing the stress relaxation time from 10 to 40 min at 25 °C. Studies have shown that the elastic springback can reach approximately zero [37,55,56]. 

### 3.3. Recovery

To induce recovery of the samples (*R_e_*), a thermal stimulus was applied in a Shimadzu TCE-N300 thermostatic chamber. The recovery temperature was set at 60 °C (5 °C + Tg). To ensure a consistent temperature throughout the chamber, the temperature was maintained at 60 °C for 10 min before the samples were placed inside. The samples remained in the chamber for 1.5 h to achieve complete recovery. After removing the samples from the chamber, a short cooling period was allowed at room temperature. Then, the recovery height (*H_re_*) was measured. The recovery value (*R_e_*) was obtained by subtracting *H_es_* from *H_re_* (Equation (2)).

(2) $$R_{e}\;\left(mm\right)=H_{re}\left(mm\right)-H_{es}\;\left(mm\right)$$

**Figure 5:**
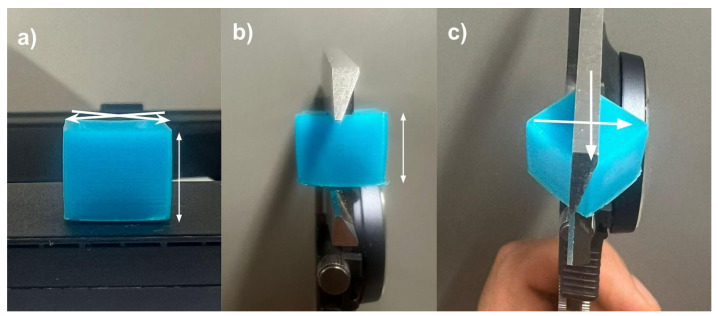
(**a**) Positions for height measurements, (**b**) one in the middle of the sample (white arrow), (**c**) two for the opposite corners (white arrows).

To account for potential warping, the recovered sample heights were determined by averaging three measurements: one at the center (*z*-axis) and two at opposite corners relative to the reference plane (Figure 5). A Mitutoyo^®^ Vernier caliper was used for these measurements.

Table 3 shows the values obtained for the recovery of the different displacements for both patterns. The recovery tended to increase as the displacement increased. The maximum recovery was 3.68 mm for the criss-cross pattern at 5.7 mm of displacement. 

At 60 °C, the material’s unstable structure from compression reverted to its original configuration. This recovery process caused the material to contract along the direction in which the filament was deposited (raster) and expand perpendicularly. Microscale damage from plastic deformation can also be partially repaired [37]. Internal stresses drive this expansion, enabling material recovery. Although the measured recovery showed no significant difference between the IPs, the order of the filament deposition (infill strategy) and the bonding between them can still limit recovery. These factors can influence whether the filaments act as a cohesive unit (coupled) or independently (noncoupled).

### 3.4. Shape Fixity and Recovery Ratios

The SFR considers how much deformation is maintained after a load is removed at the end of compression, and it was calculated considering *E_s_* divided by *D_d_* and substituted in Equation (3).

(3) $$\%\;SFR=\left(1-\frac{\;E_{s}\left(mm\right)}{D_{d}\left(mm\right)}\right)\;*\;100$$

The SRR considers how much deformation is recovered after the application of the stimulus, and it was calculated considering *R_e_* divided by *D_d_*. These values were substituted into Equation (4).

(4) $$\%\;SRR=\frac{R_{e}\;\left(mm\right)}{D_{d}\left(mm\right)}\;*\;100$$

**Figure 6:**
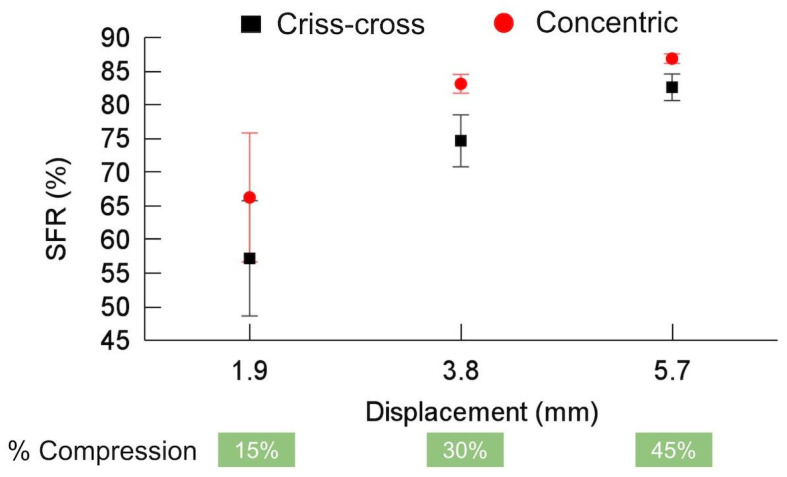
Shape fixity ratio (SFR) for criss-cross and concentric infill patterns.

Figure 6 shows the SFR of the criss-cross and concentric patterns. The maximum SFR was ~87% for the concentric pattern at 5.7 mm of displacement. As the displacement increased, the SFR also increased, since the influence of the elastic springback relative to the total deformation decreased at higher strains. Other authors have observed that increasing the displacement or strain can improve the SFR [40]. As the displacement increased, the SFR tended to be similar for both patterns. The obtained SFRs are similar to those reported by other authors (~80%) [37,40], particularly at the maximum displacement [37]. Shahi et al. [37] decreased the elastic springback and consequently improved the SFR from 89.32% to 95.90% when the stress relaxation time was increased from 10 to 40 min at 25 °C. Studies have shown that the elastic springback can reach approximately zero (SFR ~ 100%) without considering changes in AM PPs [37,55,56].

Compared to other programming methods (HP and WP), CP has the lowest SFR because the proportion of moving segments within the polymer structure decreases with decreasing programming temperature, while the intermolecular frictional barriers increase [40]. The limited number of moving segments and absence of free volume contraction in the surroundings make the entropic force storage less effective, resulting in a lower SFR [40].

It should be noted that the applied strains for CP are derived from elastic, plastic, and viscoelastic strains; the elastic component cannot be saved and returns after unloading regardless of time because it arises from segments that have relatively weak constraints or a large free volume around them. The viscoelastic and plastic components are time-dependent and may be released over time due to their constraints and rigid boundary conditions [40]. According to [40], in CP, the sample is in a glassy state, and segmental motions and virtual space for conformational changes are limited. These limited suitable sites for segmental conformational changes undergo heterogeneous conformational changes at high concentrations, leading them to exhibit considerable elastic springback within an enlarged free volume, influencing the SFR. 

**Figure 7:**
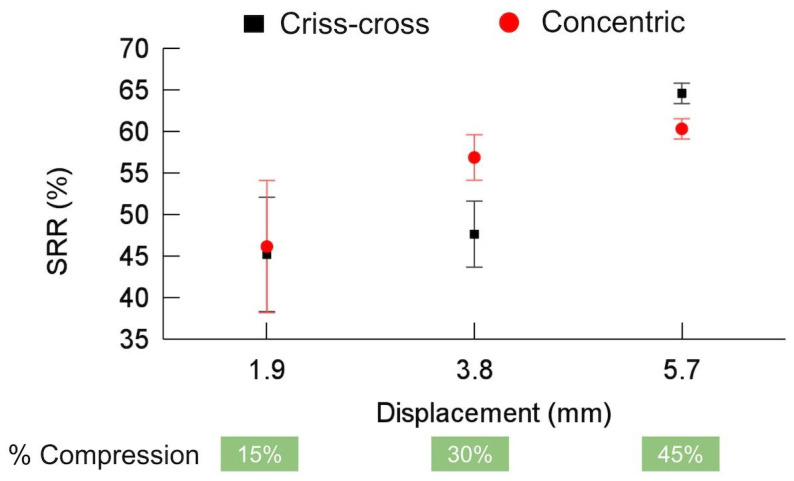
Shape recovery ratio (SRR) for criss-cross and concentric infill patterns.

Figure 7 presents the SRR for both IPs. The criss-cross pattern achieved a maximum SRR of approximately 65% at a displacement of 5.7 mm. Interestingly, the SRR for the concentric pattern initially increased faster with displacement but remained marginally lower than that for the criss-cross pattern at 5.7 mm. Overall, both patterns exhibited a trend of an increasing SRR as the displacement increased.

The obtained SRRs are lower than those reported by other authors, which are almost 100% considering CP [40,46,50,51]. Li and Wang [54] suggested that as temperature increases, some frozen internal space (free volume) is gradually released, constraints are reduced, and the locked stress or energy is released. The molecules or segments under strong constraints gradually transform from a nonequilibrium configuration to an equilibrium configuration, leading to stress or shape recovery. Recovery can occur at a lower temperature but over a longer time [54].

### 3.5. Maximum Recovery Force

A constrained recovery test was performed to determine the maximum recovery force (N). A similar procedure was followed for the recovery of the samples, but the samples were placed between the claws of the tensile tester after 10 min to ensure temperature homogeneity inside the Shimadzu Autograph AGS-X 50 kN Precision Universal Tester thermal chamber in compression mode at 60 °C to apply the stimulus. The data were collected with TRAPEZIUM LITE X software (version 1.5.0c). The software was programmed to realize a controlled compression test with a load cell of 5 kN. A preload of 32 N was applied to the samples to ensure contact and avoid movement between the plates at 5 mm/min. Then, a hold of 30 min was considered to ensure total energy release. Subsequently, the plates were returned immediately to the initial position.

**Figure 8:**
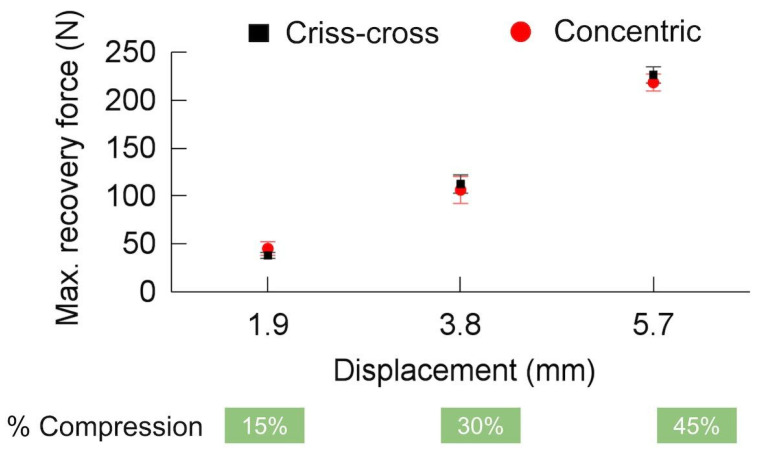
Maximum recovery force for criss-cross and concentric infill patterns.

The maximum recovery force was determined from the peak value recorded during the test. As shown in Figure 8, the maximum recovery force increased for both IPs with increasing compression percentage. Interestingly, both patterns exhibited very similar maximum recovery forces (approximately 220 N, equivalent to 1.4 MPa).

Previous research [37,45] has suggested that the recovery stress originates from the stored energy of the polymer as it attempts to return to its original shape. This stored energy tends to be greater for polymers programmed at lower temperatures. In the context of this study, the higher loads used for CP compared to WP and HP [37] likely contributed to the observed phenomenon. However, it is important to note that higher programming loads can also increase the possibility of damage within the samples, potentially leading to reduced recovery stresses [54]. The mechanism behind CP involves increasing the internal energy through increased potential energy, with minimal free volume contraction [40].

## 4. Conclusions

In this study, the influence of infill pattern (criss-cross and concentric) on the shape memory effect (SME) of PLA cold-programmed (CP) under different displacements was investigated. The results demonstrated that while the stress–strain curves varied due to infill pattern (IP) or other process parameters, the loads required for compression cold programming (CP) of 4DAM components and the subsequent responses to thermal stimuli also varied across displacement values. As the yield point marks the initiation of the cold-programming process, the concentric pattern exhibited lower stress, strain, and energy requirements for initiation, while the shape recovery ratio (SRR) varied across displacement values. The findings also complement those of other studies where the yield point was modified by changing material, strain rate, or temperature but also by changing process parameters such as infill pattern, which consequently modify component properties and responses due to the differing structural arrangements.

The study case showed that elastic springback, shape fixity ratio (SFR), SRR, and maximum recovery force tended to increase with increasing displacement. In all cases, the highest values were obtained at the maximum displacement. This suggests the potential for further improvements at higher displacement levels, provided the fracture point is not exceeded. Future research is necessary to fully understand this phenomenon for other materials, geometries, process parameters, CP conditions, and stimuli. The mechanical properties of components intended for 4DAM and cold programming must be carefully considered.

## Figures and Tables

**Table 1:** Process parameters for the manufactured cubic samples.

Process Parameters	
Material	PLA
Filament diameter (mm)	1.75
Extrusion temperature (°C)	210
Bed temperature (°C)	45
Layer height (mm)	0.20
Nozzle diameter (mm)	0.40
Deposition speed (mm/s)	30
Infill density (%)	100
Infill pattern	Criss-cross (−45/45) Concentric

**Table 2:** Elastic springback of criss-cross and concentric infill patterns.

Displacement (mm)	Elastic Springback (mm)	
	Pattern	
	Criss-cross	Concentric
1.9	0.81 ± 0.16	0.64 ± 0.18
3.8	0.96 ± 0.15	0.64 ± 0.05
5.7	0.99 ± 0.11	0.75 ± 0.04

**Table 3:** Recovery of criss-cross and concentric infill patterns.

Displacement (mm)	Recovery (mm)	
	Pattern	
	Criss-cross	Concentric
1.9	0.86 ± 0.13	0.88 ± 0.15
3.8	1.81 ± 0.15	2.16 ± 0.10
5.7	3.68 ± 0.07	3.44 ± 0.07

## Data Availability

The original contributions presented in this paper are included in the article; further inquiries can be directed to the corresponding author.
